# Modeling opioid overdose events recurrence with a covariate-adjusted triggering point process

**DOI:** 10.1371/journal.pcbi.1012889

**Published:** 2025-05-05

**Authors:** Fenglian Pan, You Zhou, Carolina Vivas-Valencia, Nan Kong, Carol Ott, Mohammad S Jalali, Jian Liu

**Affiliations:** 1 Department of Systems and Industrial Engineering, University of Arizona, Tucson, Arizona, United States of America; 2 Weldon School of Biomedical Engineering, Purdue University, West Lafayette, Indiana, United States of America; 3 Department of Biomedical and Chemical Engineering, The University of Texas at San Antonio, San Antonio, Taxes, United States of America; 4 Edwardson School of Industrial Engineering, Purdue University, West Lafayette, Indiana, United States of America; 5 College of Pharmacy, Purdue University, Indianapolis, Indiana, United States of America; 6 MGH Institute for Technology Assessment, Harvard Medical School, Boston, Massachusetts, United States of America; Yale School of Public Health, UNITED STATES OF AMERICA

## Abstract

Substance use disorder, particularly opioid-related, is a serious public health challenge in the U.S. Accurately predicting opioid overdose events and stratifying the risk of having such an event are critical for healthcare providers to deliver effective interventions in patients with opioid overdose. Despite a large body of literature investigating various risk factors for the prediction, the existing research to date has not explicitly investigated and quantitatively modeled how an individual’s past opioid overdose events affect future occurrences. In this paper, we proposed a covariate-adjusted triggering point process to simultaneously model the effect of various risk factors on opioid overdose events and the triggering mechanism among opioid overdose events. The prediction performance was assessed by the U.S. state-wise Medicaid reimbursement claims data. Compared with commonly used prediction models, the proposed model achieved the lowest Mean Absolute Errors and Mean Absolute Percentage Errors on 30-, 60-, 90, 120-, 150-, and 180-day-ahead predictions. In addition, our results showed the statistical significance of considering the triggering mechanism for recurrent opioid overdose events prediction. On average, around 47% of the event recurrence were explained by the triggering mechanism.

## Author summary

In complex natural systems, discrete event sequences often occur stochastically, such as earthquakes, financial transactions, and drug overdoses. The occurrence of these events is typically influenced by various factors (e.g., the occurrence of earthquakes is influenced by tectonic zones experiencing deformation). Given these known factors, however, such events do not appear independently. Instead, the occurrence of one event additionally influences the rate of subsequent events (e.g., a mainshock increasing the rate of subsequent aftershocks). A similar pattern can be observed in drug overdose events, where occurrences are related to various risk factors, and an just occurred overdose increases the risk of subsequent overdoses. Existing methods that identify associations between overdoses and the risk factors provide limited insight into inter-event dependencies. Recently, the triggering event point process has been used to capture these complex inter-event dependencies. In this study, we propose a covariate-adjusted triggering point process, extending the conventional models to simultaneously account for the impacts of various factors and inter-event dependency on event occurrence. The results of the real data analysis demonstrates that proposed model has the potential to provide unique insights into the nature of inter-event dependencies and significantly improve prediction accuracy.

## Introduction

The United States (U.S.) is experiencing a nationwide opioid overdose epidemic, which witnesses a noticeable mortality rate and socioeconomic burden [1]. This situation has been exacerbated in recent years due to the emergence of fentanyl in the illicit opioid market since 2013 [2]. In 2020, opioids were involved in about 70,000 overdose deaths in the U.S. [3], and this number rose to over 80,400 in 2021 [4]. Opioid overdose is the most common cause of death among various substance overdoses. For example, it accounted for 81.7% of the total drug overdose deaths in 2016 [5]. In response to this crisis, the U.S. Centers for Disease Control and Prevention (CDC) has added opioid overdose prevention to its list of top five public health challenges in 2014 [6].

In this context, identifying individuals at high risk of opioid overdose has been shown as a key opportunity to connect patients with essential services for potentially lifesaving prevention [7]. In addition, predicting the temporal trends of recurrent opioid overdoses may help evaluate the impact of prevention efforts, particularly given the predominance of fentanyl and the exacerbation of the overdose crisis by the COVID-19 pandemic [8]. In this paper, we aim to establish a general statistical model to model the intensity of recurrent opioid overdose events for each patient, which can be viewed as the instantaneous rate of opioid overdose occurrence over time, is an important indicator of the risk of opioid overdose. Based on the estimated intensity, we can predict the risk of opioid overdose and the temporal trends of the recurrent opioid overdoes in the future time intervals.

**Fig 1:**
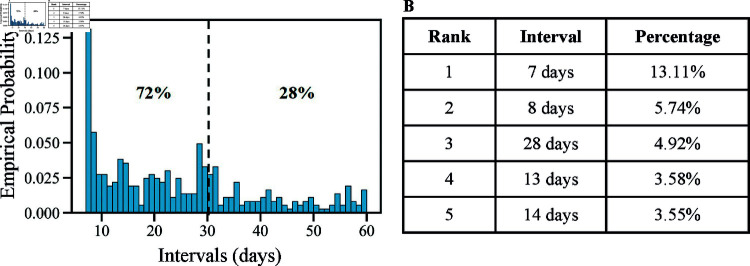
Distribution of time intervals between two consecutive opioid overdoses. (A) Histogram of the time intervals. (B) Top 5 time intervals.

While understanding and predicting the occurrence of opioid overdoses is crucial, it faces some challenges due to the complex opioid overdose occurrence processes. First, anyone with opioid use disorder (OUD) may experience an opioid overdose at any moment randomly, and the intrinsic tendency of having an opioid overdose can differ significantly from one patient to another. This difference may be related to various risk factors, including age, sex, substance amount used, and treatment received [9]. Second, opioid overdose often occurs recurrently in the majority of patients who suffer from chronic OUD [10]. Given all of the known risk factors, the recurrent overdose events may not be independent. With the chronic condition, an already occurred opioid overdose may trigger the occurrence of subsequent opioid overdoses, which is regarded as an event-to-event triggering mechanism in this paper. The event-to-event triggering mechanism exists in biobehavioral health, making opioid overdose recurrence history-dependent and influencing the occurrence rate of subsequent opioid overdoses. Intuitively, this is because a patient with OUD consumes an excessive amount of opioids, leading to an opioid overdose, which could create a pattern of repeated opioid use and recurring opioid overdoses in the future. Empirically, we explored the distribution of time intervals between two consecutive opioid overdoses among recipients in our data. As shown in Fig 1, around 72% of time intervals between two consecutive opioid overdoses are within thirty days. This also suggested that an individual who has experienced an overdose is at a high risk of subsequent overdoses in the near future, aligning with the finding in the existing literature [11,12]. Our research was motivated by the need to understand and quantify the additional event-to-event triggering mechanism among overdoses, apart from effects captured by the known risk factors. This work is enabled by the opportunities and challenges in analyzing opioid overdose records among Medicaid recipients, using reimbursement claims data collected by a state Medicaid office.

In the literature, considerable methods, including clinical assessment rules, statistical models, and machine learning (ML) and deep learning (DL) methods, have been developed to examine risk factors that are associated with opioid overdose, such as demographic factors [9] and clinical characteristics [13]. For example, at the early stage, clinical assessment criteria were proposed to identify patients at risk by examining whether high-risk criteria (e.g., need for intubation and seizures) were present in a patient’s medical record (e.g., emergency room notes, progress notes by doctors and nurses, and vital signs sheets) [14]. Subsequently, clinical prediction rules were developed to synthesize potentially influencing clinical characteristics (e.g., blood pressure and comorbid illnesses) associated with overdose [15]. These clinical prediction rules are knowledge-based and generally rely on limited clinical test data. Normally, they can only make reasonable predictions on high-risk encounters *within one event episode*. This is unlikely applicable to our prediction problem, which studies the tendency and the recurrence of opioid overdoses.

With increasing data available, statistical models, including survival analysis [16] and Poisson regression-based models [17], have emerged in recent years for understanding the quantitative relationship between opioid overdose and various risk factors. Such methods model the risk of opioid overdose as a function of risk factors in six categories, including patient’s demographics, mental health comorbidities, substance use disorders, physical health comorbidities, characteristics of opioids prescribed, and non-opioid medications prescribed [9]. For instance, the relationship between the risk of opioid overdose and the risk factors can be represented as a hazard function in a Cox Proportional Hazards Regression (CPHR) [18]. A five-variable CPHR (i.e., age, age-squared, long-acting and extended-release formulation, tobacco use, and mental health and substance abuse/dependence diagnoses) was developed to identify high-risk opioid overdose individuals [16]. However, conventional CPHR-based models are mainly designed to model survival data, referring to a type of data where the primary outcome of interest is the time until the event of interest (e.g., death or disease diagnosis) occurs. They only take into account the first overdose occurrence and discard subsequent event information, which likely leads to modeling inaccuracy due to interdependence between the recurrent events.

To analyze recurrent events, the Andersen-Gill (A-G) model [19] that generalizes CPHR was proposed to allow for the analysis of recurrent event data. For example, a multivariate A-G model was used to identify demographic, health, social, and criminal justice predictors of non-fatal overdose [20,21]. The Poisson regression models were also widely used in modeling recurrent event data, which focus on the number of times that an event of interest has occurred [22]. For instance, log-linear and logistic Poisson models with a Bayesian space-time framework were used to predict the future course of overdose mortality [17]. A Poisson regression model was used to examine the relationship between prescription opioid rates and prescription opioid overdose deaths using spatial cluster and regression analyses [23]. Such models are specially designed to count data and assume that the opioid overdoses are independent. Thus, they cannot model the event-to-event triggering mechanism among recurrent events.

Recently, the development of interoperable health IT technology and the establishment of large data repositories with sufficient data harmonization has helped realize longitudinal environments [24]. For example, a large quantity of longitudinal data related to overdose, including hundreds of potential risk factors, were acquired from electronic health records (EHRs) [25]. With such data readily available, multiple ML techniques, including random forest [26], decision tree [1], logistic regression [27], and *K*-means [28] were used to predict an individual’s risk of overdose. In addition, deep learning methods gained popularity for EHR-based predictive modeling. A long short-term memory (LSTM) model was built to identify whether a patient is at high risk of overdose in the next hospital visit [29]. These studies have demonstrated that ML- and DL-based predictive models can achieve promising results in learning a patient’s risk of opioid overdose from EHR history. However, there are two main differences between the existing ML- and DL-based method and our proposed method. First, our method enables reliable identification and quantification of the triggering mechanism, which is not explicitly considered in the existing methods. Second, our research focuses on predicting the occurrence rate of future recurring opioid overdoses. The classification-based methods focus on determining whether a patient faces a high risk of opioid overdose in the future.

The proposed method is based on the Hawkes process [30], which is a special type of point process and models self- and mutual-exciting patterns among recurrent events. It has drawn much attention in various fields, such as seismology [31], criminology [32], epidemiology [33], neural activity [34], and engineering [35]. The intensity function of the Hawkes process consists of two terms: a baseline intensity, which is independent of past events, and a triggering intensity, which captures the triggering effects of past events on future events. For example, each earthquake usually triggers a sequence of aftershock activities, and the occurrence rate of aftershocks can be represented in the triggering intensity function. The self- and mutual-exciting point processes align well with the aforementioned event-to-event triggering mechanism among recurrent opioid overdoses. Motivated by the need of understanding the additional triggering mechanism among overdose events and better predicting the occurrence of overdose events, we propose a covariate-adjusted triggering point process to model the intensity function of recurrent opioid overdoses. Specifically, the intensity function is controlled by two components: the baseline event rate, which is used to capture the heterogeneity of individuals such as demographics factors, and is independent of past opioid overdoses, and the triggering intensity, which is collected from the positive triggering effect of every past opioid overdoses.

The rest of this paper is organized as follows. Methods section introduces a methodological framework that includes study cohorts, model formulation, and model estimation. Results section presents the model implementation and demonstrates the advantages of our proposed method in terms of model prediction accuracy and interpretability. The conclusion and future work will be discussed in Discussion section and the Limitations section summaries the limitations of our methods.

## Methods

### Ethics statement

This study was approved by the Purdue University Institutional Review Board (2019-118). The IRB was an umbrella IRB that was used by a recently awarded grant from the United States Centers for Medicare and Medicaid (CMS) through the Indiana Family and Social Service Administrations (FSSA) so all Purdue researchers were able to utilize the data for their studies. Co-authors Kong and Vivas-Valencia (then as a PhD student at Purdue) were included on the original IRB. Co-author Zhou was added once he became a graduate student at Purdue in 2021. The data was physically transferred to Purdue University and housed on a secured data server. The data was further de-identified by a centrally appointed data management technician. Then only the de-identified data were made available to the Purdue campus-wide researchers. The data used for our analyses reported in this manuscript were prepared by Vivas-Valencia under the guidance of co-authors Kong’s and Ott’s. The reporting of Medicaid data by health care providers was mandated by the United States Centers for Medicare and Medicaid (CMS) for all Medicaid beneficiaries. Because of the de-identified nature of the data, we did not own the data and none of the authors were designated custodian of the data for data sharing with Purdue. At any point of the research project, non-Purdue researchers were granted the access to the data set. The workflow was such that the University of Arizona researchers prepared the codes based on the discussion about the data structure and they sent the code to Purdue so co-author Zhou could run the code on the de-identified data.

### Cohort selection, index date, and covariates

**Fig 2:**
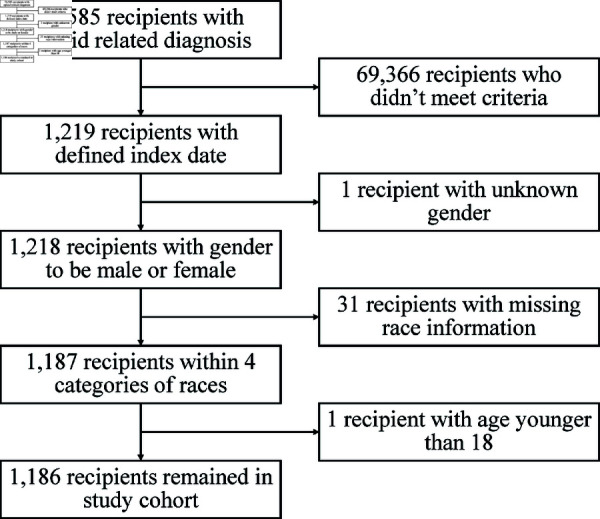
Flowchart of data extraction and preprocessing.

We identified recipients enrolled in the a state-wise Medicaid program who experienced two or more opioid overdoses between January 1, 2016, and December 31, 2018. Opioid overdoses were determined using ICD-10-CM diagnosis codes (see supplementary material for the list of codes). For each patient, we recorded the earliest overdose date following a baseline period of 90 days with no observed overdoses. The date 90 days after the earliest overdose date was defined as the individual’s *index date*. Patients were excluded from the study if there were no overdoses after the index date. This is because individuals with only one recorded overdose do not include recurrence information. Additionally, our dataset only recorded overdoses between January 1, 2016, and December 31, 2018. For individuals with only one recorded overdose, it is unclear whether this reflects a genuinely single event or a truncated record of their history. Including individuals assumed to have only one overdose (but who might have experienced additional overdoses outside the observation period) could introduce bias into the model estimation. Patients were excluded from the study if they were under 18 years old on the index date or had missing demographic information. A detailed flowchart of the data extraction and preprocessing process is shown in Fig 2. After preprocessing, we identified 1,186 patients who met these criteria. These patients were followed from the index date until they were censored on December 31, 2018. The model was trained on 949 patients (80%) and externally validated in the remaining 237 patients (20%).

We included six known covariates that may influence the baseline intensity of opioid overdoses, including demographics, opioid withdrawal, and medications for opioid use disorder (MOUD). For demographic information, we considered the following categorical variables: (i) sex, classified as male or female; (ii) age, grouped into young adults (18-34), middle-aged adults (35-49), older adults (50-65), and seniors (66+); (iii) race, categorized into four groups: Caucasian, Black, Hispanic, and Others; and (iv) geographic classification, where the state was divided into rural, urban, and mixed rural-urban areas based on 3-digit ZIP codes. These demographic covariates were observed at the start of the study and were treated as static covariates over the observation period. The opioid withdrawal and MOUD were treated as time-dependent binary variables in our model.

### Model formulation

The stochastic nature of randomly recurring opioid overdoses can be modeled as a point process, where the recurring opioid overdoses are treated as a series of random points distributed in time [36]. Consider a data set that includes the opioid overdoses recorded from a cohort of *N* recipients, in which each recipient *i* , *i* = 1 , ⋯ , *N*, can experience opioid overdoses recurrently during the observed window  [ 0 , *T* ) . Let $t_{i}$ denote a collection of timestamps of opioid overdoses of recipient *i*, i.e., $t_{i}=\{t_{ij}|j=1,\cdots \;,n_{i}\}$, where $t_{i1}<\cdots <t_{in_{i}}$ and $n_{i}$ denotes the total number of opioid overdoses of recipient *i*. In addition to the timestamps of opioid overdoses, this paper considers the recipient’s demographic factors associated with the opioid overdoses, such as sex, age, and race. Let $x_{i}\in X\subset {\mathbb{R}}^{p}$ denote a vector of demographic factors of recipient *i*. Thus, the historical data of recipient *i* during  [ 0 , *T* ) , can be denoted as $H_{i}(T)=(t_{i},x_{i})$ and the overall historical data from *N* recipients, during  [ 0 , *T* ) , can be denoted as $H(T)=\{H_{i}(T)|i=1,\cdots \;,N\}$.

From a modeling point of view, the opioid overdoses of recipient *i*, i.e., $t_{i}$, can be treated as a series of random points occurring in time  [ 0 , *T* )  and be modeled by a point process. Specifically, for each recipient *i*, its expected instantaneous occurrence probability of an opioid overdose at time *t* can be modeled by a conditional intensity function (CIF, “*intensity*" hereafter) given a set of historical event data, $H_{i}(t)$, during  [ 0 , *t* ) , i.e.,


λi(t|Hi(t))= lim ⁡ dt→0E[N([t,t+dt))|Hi(t)]∕dt, (1)

where $N([t_{1},t_{2}))$ denotes a non-negative integer-valued point process counting the number of opioid overdoses occurring in the time interval $\left[t_{1},t_{2}\right)$, and $E[N([t,t\;+\;dt))|H_{i}(t)]$ is the expected number of opioid overdoses occurring in the time interval  [ *t* , *t* + *dt* ) , given $H_{i}(t)$.

As discussed in Introduction section, each recipient using opioids may experience an opioid overdose at any moment randomly, and the probability of having such an opioid overdose may be related to various risk factors or the event-to-event triggering mechanism. Such occurrence characteristics of opioid overdose provide us a hint to decompose the total intensity of opioid overdoses into two parts. Specifically, for each recipient *i*, an opioid overdose may be caused by various risk factors, whose occurrence randomness is modeled by a baseline intensity $\lambda_{i}^{b}$, with the superscript “*b*” denoting a baseline intensity, or it may be triggered by an occurred opioid overdose, which can be modeled as $\lambda_{i}^{t}$, with the superscript “*t*" denoting the triggering intensity, and be combined with the baseline intensity function, $\lambda_{i}^{b}$. Thus, the overall opioid overdose occurrence intensity of recipient *i* can be formulated as:


$$\begin{array}{ccc} \lambda_{i}(t|H_{i}(t))=\lambda_{i}^{b}(t)+\lambda_{i}^{t}(t),i=1,2,...,N, & & \end{array}$$ (2)

where $H_{i}(t)$ represents historical data of recipient *i*. It is worth noting that there is no label indicating whether an overdose is due to risk factors or triggered by a specific mechanism.

#### Baseline intensity.

It has been shown that the risk of opioid overdose is time-varying [37] and related to various risk factors, such as demographics and health conditions. For example, evidence shows that opioid dosage was the factor most consistently associated with increased risk of opioid overdose [38]. In this context, we model the baseline intensity, $\lambda_{i}^{b}(t)$, in a log-linear form, as often used in Cox-type regression models in public health [39,40], i.e.,


$$\begin{array}{ccc} \lambda_{i}^{b}(t)=\lambda_{0}(t)\cdot \exp(\alpha^{⊤}x_{i}), & & \end{array}$$ (3)

where $\lambda_{0}(t)$ is a function used to model the temporal dependencies of baseline rate. It can be time-independent or time-dependent. Without loss of generality, we adopt a Power Law function, i.e., $\lambda_{0}(t)=\theta \;\cdot \;t^{\theta -1}$, which is mathematically simple and flexible to capture the constant (when *θ* = 1), decreasing (when *θ* < 1), and increasing (when *θ* > 1) event rates. Additionally, model selection was performed using the Akaike Information Criterion (AIC) and Bayesian Information Criterion (BIC). The results demonstrated that the model with one-parameter Power Law function outperformed the model with three-parameter Weibull function, as it achieved lower AIC and BIC values. In addition, we incorporate individualized covariates $x_{i}\in \theta \subset {\mathbb{R}}^{p}$ into the baseline intensity with a log-linear form with the corresponding coefficient vector $\alpha \in A\subset {\mathbb{R}}^{p}$, to model the heterogeneity of individuals.

#### Triggering intensity.

In addition, an occurred opioid overdose may trigger the occurrence of subsequent opioid overdoses. The existence of the event-to-event triggering mechanism additionally increases the intensity of future opioid overdoses to the baseline intensity. Motivated by Hawkes process [30], this paper models the increased intensity, i.e., triggering intensity, $\lambda_{i}^{t}(t)$, as:


$$\begin{array}{ccc} \lambda_{i}^{t}(t)=\underset{j:t_{ij}<t}{\sum }\beta \cdot g(t-t_{ij}), & & \end{array}$$ (4)

where the term $\beta \cdot g(t-t_{ij})$, *β* > 0 represents the triggering effect on overdose intensity at time *t* caused by a past overdose occurring at time $t_{ij}$. Specifically, *β* quantifies the magnitude of the increase in overdose intensity following an overdose at $t_{ij}$, which subsequently decays according to the decay function *g* ( ⋅ )  until the next overdose occurs to raise the intensity again. In this study, without loss of generality, we employed an exponential decay function, *g* ( *t* ) = *γ* ⋅ *exp* ⁡  ( − *γ* ⋅ *t* ) , *γ* > 0. This function was chosen for its simplicity and effectiveness in capturing the natural decay of the triggering effect over time. It has been well-established and widely applied in fields such as epidemiology [33] and health analytics [41].

With the above specification, the total intensity function of recipient *i* in Eq (2) is expressed as:


$$\begin{array}{ccc} \lambda_{i}(t|H(t))=\theta \cdot t^{\theta -1}\cdot \exp(\alpha^{⊤}x_{i})+\underset{j:t_{ij}<t}{\sum }\beta \cdot \gamma \cdot \exp(-\gamma \cdot (t-t_{ij})). & & \end{array}$$ (5)

With parameters *Θ* = { *θ* , *α* , *β* , *γ* }  in Eq (5) estimated, we can calculate the cumulative intensity of opioid overdose (also known as the cumulative hazard) by $\Lambda_{i}(t)=\int_{0}^{t}\lambda_{i}(u)du$ for recipient *i*. Thus, the risk of opioid overdose by time *t* can be calculated by $1-\exp(-\Lambda_{i}(t))$ [42].

### Model estimation

This paper uses the maximum likelihood estimation (MLE) method for parameter estimation. Let *T* denote the length of the observation window, given the historic observational data $H(T)=\{(t_{i},x_{i})|i=1,...,N\}$, the likelihood function associated with overall parameters *Θ* = { *θ* , *α* , *β* , *γ* }  defined in Eq (5), can be formulated as,


$$\begin{array}{ccc} L(\Theta )=\overset{N}{\underset{i=1}{\prod }}\{\overset{n_{i}}{\underset{j=1}{\prod }}\lambda_{i}(t_{ij};x_{i},\Theta )\}\times \exp\{-\Lambda_{i}(T;x_{i},\Theta )\}, & & \end{array}$$ (6)

where the intensity function $\lambda_{i}(t;x_{i},\Theta )$ is defined in Eq (5), and the term $\Lambda_{i}(T;x_{i},\Theta )=\int_{0}^{T}\lambda_{i}(t;x_{i},\Theta )dt$ is the cumulative intensity function. The log-likelihood function is obtained by taking the logarithm of *L* ( *Θ* )  in Eq (6). That is,


$$\begin{array}{ccc} \ell (\Theta )=\overset{N}{\underset{i=1}{\sum }}\{\overset{n_{i}}{\underset{j=1}{\sum }}log(\lambda_{i}(t_{ij};x_{i},\Theta ))\}-\Lambda_{i}(T;x_{i},\Theta ), & & \end{array}$$ (7)

where the first term depends on the historical observation data, i.e., $H(T)=\{(t_{i},x_{i})|i=1,\cdots \;,N\}$ and can be easily computed by plugging the historical data into Eq (5). The second term is a definite integral of Eq (5), which can be explicitly expressed and depends on observed demographic factors, i.e., $x_{i},i=1,\cdots \;,N$. Thus, the MLE of *Θ*, denoted by $\hat{\Theta }$, can be obtained by finding the value of *Θ* that maximizes *ℓ* ( *Θ* )  in Eq (7), i.e.,


$$\begin{array}{ccc} \Theta =argmax_{\Theta }\ell (\Theta ). & & \end{array}$$ (8)

To address the bounded constraints on some of the parameters in *Θ*, we use the “L-BFGS-B” option in the Python minimize() function. The “L-BFGS-B” algorithm allows users to specify an interval for each variable to be optimized [43].

## Results

### Model implementation

We applied the proposed method to the study cohort. The cohort was randomly divided into a training set, comprising 80% of the data, and a test set, comprising the remaining 20%, with 100 replications. In each replication, *r*, the proposed model was trained based on the training set to obtain the estimated parameters ${\hat{\Theta }}^{r}$ and predicted intensity ${\hat{\lambda }}_{i}^{r}(t,{\hat{\Theta }}^{r})$. Given ${\hat{\lambda }}_{i}^{r}(t,{\hat{\Theta }}^{r})$, the model was externally validated in the test set. Specifically, the number of opioid overdoses in the *M* recipients in the test set during time interval  [ 0 , *L* )  can be predicted as [42]:


$$\begin{array}{ccc} {\hat{m}}_{\left[0,L\right)}^{r}=\overset{M}{\underset{i=1}{\sum }}\int_{0}^{L}{\hat{\lambda }}_{i}^{r}(t,x_{i},{\hat{\Theta }}^{r})dt, & & \end{array}$$ (9)

where *L* is the length of the prediction window. The predicted number above would be compared to the actual number of opioid overdoses in the test set for the same time interval, denoted as $m_{\left[0,L\right)}^{r}$. For a certain length of the prediction window, *L*, this comparison was conducted for 100 times, i.e., *r* = 1 , 2 , . . . , 100.

### Model evaluation

To measure the prediction accuracy, we define the mean absolute error (MAE) over the *R* replications as [44]:


$$\begin{array}{ccc} \text{MAE}=\frac{\overset{R}{\underset{r=1}{\sum }}|{\hat{m}}_{\left[0,L\right)}^{r}-m_{\left[0,L\right)}^{r}|}{R}. & & \end{array}$$ (10)

A smaller MAE implies better model performance. However, the MAE is sensitive to the scale of actual numbers of opioid overdoses and can not provide a standardized measure when dealing with the prediction results at different prediction windows. In response, the mean absolute percentage error (MAPE) over the *R* prediction windows is considered and calculated by:


$$\begin{array}{ccc} \text{MAPE}=\frac{100\%}{R}\cdot \overset{R}{\underset{r=1}{\sum }}\frac{\left|{\hat{m}}_{\left[0,L\right)}^{r}-m_{\left[0,L\right)}^{r}\right|}{m_{\left[0,L\right)}^{r}}. & & \end{array}$$ (11)

MAPE is expressed as a percentage, making it more interpretable for modelers who may not know the magnitude of actual opioid overdoses. Both MAE and MAPE were used to evaluate the performance of the proposed method.

To investigate the advantage of the proposed method that explicitly considers the event-to-event triggering mechanism, we compared the prediction performance of the proposed method and the benchmarks that ignore the triggering mechanism. The benchmarks and the proposed method are listed below:

- **Homogeneous Poisson Process (HPP)** assumes that opioid overdoses of each individual are independent, and the occurrence rate is constant, i.e., the intensity of subject *i* is $\lambda_{i}(t)=\theta ,\theta >0$.
- **Non-homogeneous Poisson Process (NHPP)** assumes that opioid overdoses of each individual are independent, and the occurrence rate follows a predefined time-varying function [45]. In this paper, the adopted intensity of subject *i* is: $\lambda_{i}(t)=\theta \;\cdot \;t^{\theta -1}\;\cdot \;\exp(\alpha^{⊤}x_{i})$, where $x_{i}$ denotes the individualized covariates.
- **Andersen-Gill (A-G) model** allows for the analysis of recurrent event data [19].
- **The proposed method** explicitly considers the impacts of the demographic factors and the event-to-event triggering mechanism among opioid overdoses. The intensity of subject *i* is defined in Eq (5), i.e., $\lambda_{i}(t|H_{i}(t))=\theta \cdot t^{\theta -1}\cdot \exp(\alpha^{⊤}x_{i})+\sum_{j:t_{ij}<t}\beta \cdot \gamma \cdot \exp(-\gamma \cdot (t-t_{ij}))$.

Without loss of generality, we trained different models in the same training set and predicted the number of opioid overdoses at prediction windows of 30, 60, 90, 120, 150, and 180 days. (i.e., *L* =  30, 60, 90, 120, 150, and 180 ), respectively. The MAE and MAPE defined in Eq (10)–(11) were used to evaluate the accuracy of the prediction.

### Model accuracy

**Fig 3:**
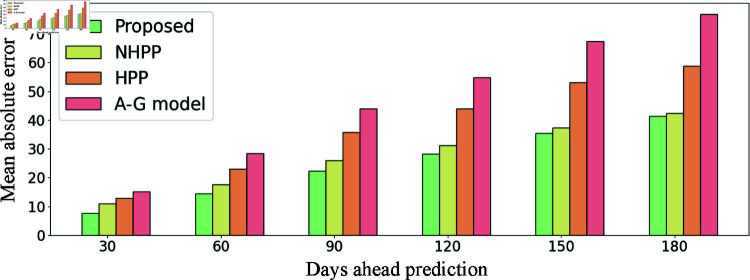
Mean absolute errors of the proposed method and the benchmarks on 30-, 60-, 90-, 120-, 150-, 180-day ahead predictions.

Fig 3 illustrated the MAEs of the proposed method and the benchmarks on 30-, 60-, 90-, 120-, 150-, and 180-days-ahead predictions, providing the average absolute differences between the true opioid overdose counts and predicted counts. There are several notable findings. First, the proposed method consistently achieves the lowest MAE across all prediction windows, highlighting its superior predictive accuracy compared to the benchmark models. Second, the MAE values increase as the prediction window lengthens, which can be attributed to the corresponding increase in true opioid overdose counts. Third, when compared to the HPP and A-G models, the proposed method reduces the MAE by a value ranging between 30%-49%, demonstrating a substantial improvement in prediction accuracy. When compared to the NHPP model, the proposed method reduces the MAE by a value ranging between 3%-30%, with performances converging for the longer prediction windows. Specifically, the proposed method lowers the MAE by 6% for the 150-day prediction window and by 3% for the 180-day prediction window.

**Fig 4:**
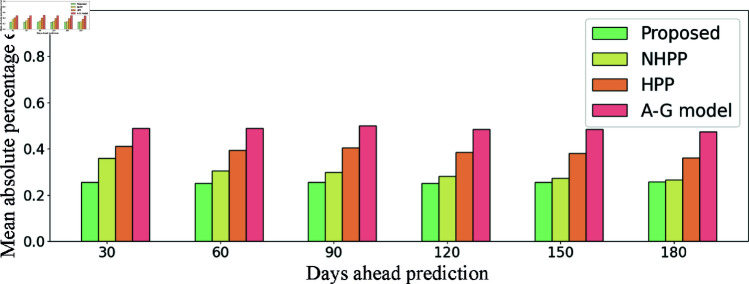
Mean absolute percentage errors of the proposed method and the benchmarks on 30-, 60-, 90-, 120-, 150-, 180-day ahead predictions.

Fig 4 displayed the MAPEs of the proposed method and the benchmarks across 30-, 60-, 90-, 120-, 150-, and 180-day prediction windows, measuring the percentage deviation between actual and predicted opioid overdose counts. The proposed method consistently achieves the lowest MAPE across all prediction windows, maintaining an approximate 25% error rate, which demonstrated its robustness in accurately predicting overdose counts.

In addition to predicting the number of opioid overdoses, we also wanted to understand and quantify the effect of the triggering mechanism on opioid overdose intensity. The quantification result can help healthcare providers predict the likelihood of some individual to have repeated overdoses and thus tailor intervention plans for these individuals based on the predicted likelihood. We first evaluated the significance of the triggering mechanism by testing whether the estimated triggering parameter, $\hat{\beta }$, across 100 repeated estimates, was significantly different from zero. Specifically, we conducted a t-test for $\hat{\beta }$ with the null hypothesis $H_{0}:\hat{\beta }=0$ and the alternative hypothesis $H_{\alpha }:\hat{\beta }>0$. The test yielded a p-value of less than 0.001. Based on our significance level, $\alpha_{c}=0.05$, we rejected the null hypothesis and included the triggering mechanism in our model.

**Fig 5:**
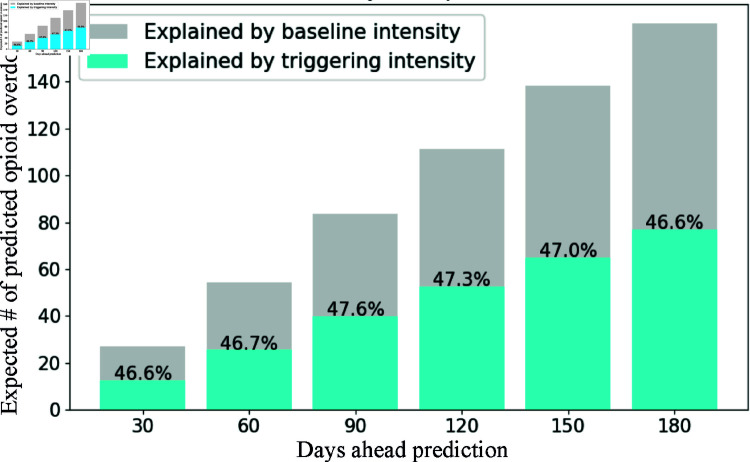
Expected opioid overdoses explained by baseline intensity and triggering intensity.

After testing the significance of the triggering effect, we calculated the proportion of the expected number of opioid overdoses explained by the triggering intensity and the baseline intensity within the test set. As shown in Fig 5, the grey bar represented the expected number of opioid overdoses explained by the baseline intensity, and the blue bar represented the expected number of opioid overdoses explained by the triggering intensity. We observed the following findings. First, different from benchmarks, the proposed method can explicitly quantify the triggering mechanism among opioid overdoses, providing a more nuanced interpretation of different types of effects. Thus, public health officials or practitioners can obtain a clearer view of the causes of opioid overdoses and assess the efficacy of interventions and policies implemented in response to the opioid crisis. Second, the proportion of the expected numbers of opioid overdoses explained by the triggering intensity consistently remained at 47% across all prediction windows, indicating that our model is robust to variations in prediction window length. Finally, the consistent 47% contribution from the triggering mechanism highlights its significant role in opioid overdose prediction.

## Discussion

Accurately predicting the risk of opioid overdose is essential for delivering effective treatment to individuals with OUD and for guiding public health policy development. The occurrence of opioid overdose is influenced not only by various risk factors but also by the event-to-event triggering mechanism inherent among opioid overdoses. This paper introduced a covariate-adjusted triggering point process that models both the underlying risk factors and the triggering effects on opioid overdose risk. For the case study, we utilized Medicaid reimbursement claims data collected from a U.S. state. The results demonstrated that the proposed method offers enhanced predictive accuracy by incorporating the triggering mechanism, which has not been considered in the existing methods. The results of our model could be used by healthcare providers to mitigate the risk of future overdoses. For example, based on the predicted risk, healthcare providers can stratify patients into higher or lower risk categories which allows for more efficient use of resources, focusing interventions on those most at risk for repeated overdoses. In addition, the proposed method allows for quantification of the triggering effect, offering a more nuanced interpretation of how different factors contribute to overdose risk, which can help healthcare providers predict the likelihood of a repeated overdose and tailor intervention plans based on individual needs.

There are several directions for our future studies. First, an immediate task will be to investigate the adaptation of the proposed method to the same data collected from other states in the U.S. Second, the proposed parametric modeling approach can be extended to allow the triggering mechanism to vary based on covariates. Third, this study primarily focused on exploring the triggering effects inherent in recurrent overdoses, which required individuals to have experienced multiple overdose events. However, in practice, some individuals may experience only a single overdose, and these cases were not included in our analysis. In the future, developing a joint modeling framework that incorporates single-overdose events, censored single-overdose events, and recurrent overdoses within a unified structure could enhance the model’s applicability to practical clinical settings.

## Limitations

While the proposed method demonstrated its good performance using Medicaid reimbursement claims data from a single state, covering the period from January 1, 2016, to December 31, 2018, several limitations should be noted. First, the study population was limited to a single state, which may reduce the generalizability of our findings to other states due to differences in drug availability, healthcare services, and prescribing patterns. Second, we were unable to account for opioid overdoses that occurred prior to January 1, 2016 and after December 31, 2018, as these data were not available. These unobserved events may have influenced subsequent overdose risk, potentially impacting our model’s results. Third, we excluded individuals with only one recorded overdose during the study period, which may introduce potential bias. This exclusion limits the generalizability of our findings to clinical settings where single-overdose patients constitute a significant proportion of the population. Fourth, Medicaid claims data may not fully capture all OUD-related events. For instance, illicit opioid use, non-fatal overdoses where medical care was not sought, or opioid prescriptions paid for out-of-pocket may not be recorded, leading to potential underestimation of overdose risk. Additionally, patients who changed insurers, relocated, or died from fatal overdoses during the study period could result in incomplete longitudinal data, which may have led to an overestimation of the risk. Unfortunately, these gaps in information could not be fully addressed. Lastly, claims data are primarily used for billing purposes and thus lack detailed clinical information and important covariates, which may have limited the predictive performance of our model.

## Supporting information

S1 Supplementary Material. **ICD-10-CM codes used to identify study population.** (PDF)

S1 Sample Data. **Data for model training and testing in Results section.** (XLSX)

S2 Sample Data Codebook. **This document serves as a reference guide for understanding sample data.** (PDF)
